# Development of an Albumin-Based PSMA Probe With Prolonged Half-Life

**DOI:** 10.3389/fmolb.2020.585024

**Published:** 2020-12-17

**Authors:** Teli Liu, Chen Liu, Yanan Ren, Xiaoyi Guo, Jinquan Jiang, Qing Xie, Lei Xia, Feng Wang, Hua Zhu, Zhi Yang

**Affiliations:** ^1^Key Laboratory of Carcinogenesis and Translational Research (Ministry of Education/Beijing), Department of Nuclear Medicine, Peking University Cancer Hospital & Institute, Beijing, China; ^2^Guizhou University School of Medicine, Guizhou University, Guiyang, China

**Keywords:** albumin, PSMA, Al^18^F, maleimidopropionic acid, Micro-PET, PRLT

## Abstract

Prostate-specific membrane antigen (PSMA) is an attractive target for the diagnosis and therapy of prostate cancer as it is specifically overexpressed in prostate cancer cells. Improving the circulation of radioligands in the blood is considered as an effective strategy that can improve tumor burden, which benefits detection of small lesions and improves the effect of PSMA radioligand therapy (PRLT). In this study, we introduced maleimidopropionic acid (MPA) to a PSMA-targeted tracer and developed Al^18^F-PSMA-CM, which targets human serum albumin (HSA) binding and PSMA. Al^18^F-PSMA-CM is evaluated *in vitro* and *in vivo* for stability, PSMA specificity, and biodistribution in 22Rv1 tumor-bearing mice. Al^18^F-PSMA-CM was prepared with a radiochemical purity of >99% and specific activity of 11.22–18.70 MBq/nmol. Al^18^F-PSMA-CM was stable *in vitro* and *in vivo* and prolonged circulation in blood with a binding ratio of 47 ± 3.2% and Kd value of 3.08 ± 0.45 nM to HSA. The uptake of Al^18^F-PSMA-CM in PSMA(+) 22Rv1 cells was increased in 2 h, and the uptake was blocked by a PSMA inhibitor, ZJ-43. The Kd value of Al^18^F-PSMA-CM to PSMA was 8.46 ± 0.24 nM. Al^18^F-PSMA-CM was accumulated in kidneys and 22Rv1 tumors [74.76 ± 15.42 and 6.16 ± 0.74 ID%/g at 2 h post injection (p.i.)], which were decreased by −80.0 and −84.3% when co-injected with ZJ-43. Al^18^F-PSMA-CM showed high PSMA specificity and accumulated in 22Rv1 tumors with increasing uptake in 4 h. MPA moiety showed the ability to prolong the half-life of tracers, and the MPA-conjugated tracer showed the potential to improve tumor uptake. MPA may be a choice to develop radiopharmaceuticals for PRLT of prostate cancer.

## Introduction

Prostate cancer (PCa) is the most common cancer in males in the world, especially in western countries. The incidence in China was low, but it increased in recent years (Siegel et al., 2017; Bray et al., 2018). Although androgen deprivation therapy (ADT) is effective for early PCa, part of patients will progress to castration-resistant PCa (CRPC) (Kirby et al., 2011). As chemotherapy and other systemic treatment options had associated toxicities or offer only a modest survival benefit, prostate-specific membrane antigen (PSMA) radioligand therapy (PRLT) has become an emerging treatment for metastatic CRPC (mCRPC) (Petrylak et al., 2004; de Bono et al., 2010; Scher et al., 2012; Parker et al., 2013).

PSMA is an attractive target for the diagnosis and therapy of PCa as it is overexpressed in most PCa and metastasis cells (Sweat et al., 1998; Rybalov et al., 2014). PSMA radioligands labeled with α or β particle emitter radionuclides (^177^Lu, ^225^Ac, etc.) can specially accumulate in PSMA-expressing tumors and act on tumor cells. The most studied radioligands were ^177^Lu/^225^Ac-PSMA-617 or ^177^Lu/^225^Ac-PSMA-I&T; they are small molecules with rapid blood clearance, which limited the achievement of therapeutic concentration in tumor tissues (Umbricht et al., 2018). In order to achieve the desirable therapeutic level, larger or more frequent doses were needed. Enhancing the blood half-life of radioligands meets the need for desirable accumulation of radioactivity in tumor cells. Some studies demonstrated that prolonging the half-life can increase the uptake of radiotracers in PSMA-expressing tumors and can show better therapeutic effects (Choy et al., 2017; Benesova et al., 2018).

One of the strategies was making use of human serum albumin (HSA); HSA acts as a versatile carrier for drug delivery due to its good biocompatibility and biodegradability, thus prolonging the active profile of fast-clearance ligands. Developing a strategy that can both conjugate a pharmaceutical and bind HSA with high efficiency and minimum side effects can effectively prolong the circulation of radioligands, which is one of the most commonly used approaches (Liu and Chen, 2016).

Three main strategies had been reported and used for binding drugs to albumin, namely, fusing the gene to the albumin gene, linking the drug with long-chain fatty acid at the binding sites of albumin, and using the bifunctional spacers to conjugate drugs to albumin.

Cysteine-34, the only free thiol of albumin in multiple species, is located in the hydrophobic crevice of albumin and is limited with reactivity, but it holds good chemical reactivity for modification to produce novel bioactive constructs with prolonged half-life. 4-(*p*-Iodophenyl) butyric acid was introduced to a PSMA inhibitor, which can bind to albumin and improve the uptake in PSMA(+) tumor and kidneys (Choy et al., 2017; Benesova et al., 2018; Umbricht et al., 2018). Evans blue is another albumin-binding molecule which has been reported to extend the half-life of tracers and shown to have higher accumulation in tumors with high ^68^Ga-PSMA-617 uptake (Zang et al., 2019). Wirtz et al. introduced bulky *p*-iodo-phenylalanine to improve the interaction of tracers and to improve the tumor uptake at 24 h post injection (p.i.). Meanwhile, the uptake in kidneys was significantly increased (Wirtz et al., 2018). Deberle et al. used isobutylphenyl propionic acid as a binding entity, which they thought can be efficiently cleared from the blood pool to keep a low background activity in healthy organs and tissues (Deberle et al., 2020).

Maleimidopropionic acid (MPA) derivatives bind to cysteine-34 of albumin by forming a thiosuccinimide bond and offer a platform for drug delivery (Gunnoo and Madder, 2016). MPA analogies have been reported to hold the ability to help drugs bind with albumin to extend their circulation half-life. Junnan et al. reported a structure, named CM, which can conjugate with albumin by intravenous injection to extend the biological half-life of peptides by 16.4 times with stronger antitumor activity (Feng et al., 2018).

In order to prepare a PSMA probe for PRLT with an appropriate half-life in the blood, we prepared a MIPA-linked PSMA ligand radiolabeled with Al^18^F to investigate the pharmacokinetics and specificity to PSMA and finally discussed the potential for PRLT of ^177^Lu- and ^225^Ac-labeled MIPA-linked PSMA tracers.

## Materials and Methods

### General

All chemicals, reagents, and solvents were purchased commercially without further purification. PSMA was purchased from Novoprotein Scientific Inc. Sep-Pak Accell Plus QMA and Sep-Pak C18 Light cartridges were purchased from Waters, and a 96-well polystyrene Stripwell™ microplate was purchased from Costar. No-carrier-added Na^18^F and Al^18^F-PSMA-BCH were provided by the Department of Nuclear Medicine, Peking University Cancer Hospital.

The product was analyzed by reversed-phase high-performance liquid chromatography (RP-HPLC; Eclipse Plus C18, 4.5 × 250 mm, 5 μm; Agilent) performed using a linear A–B gradient (15–60% of B in 15 min) with a flow of 1 ml/min. Solvents were 0.1% aqueous trifluoroacetic acid (TFA) and 0.1% TFA in acetonitrile for A and B, respectively. The HPLC system was equipped with UV and γ detectors. UV absorbance was measured at 220 nm. Nuclear magnetic resonance was performed with a Bruker Avance III HD 600 MHz spectrometer, and mass spectrometry was performed with the MALDI-MS Daltonics Microflex (Bruker Daltonics, Bremen, Germany) system. Micro-PET was performed on a Super Argus PET scanner (Sedecal, Spain).

### Chemical Synthesis

#### Synthesis of Compound A

**Scheme 1:**
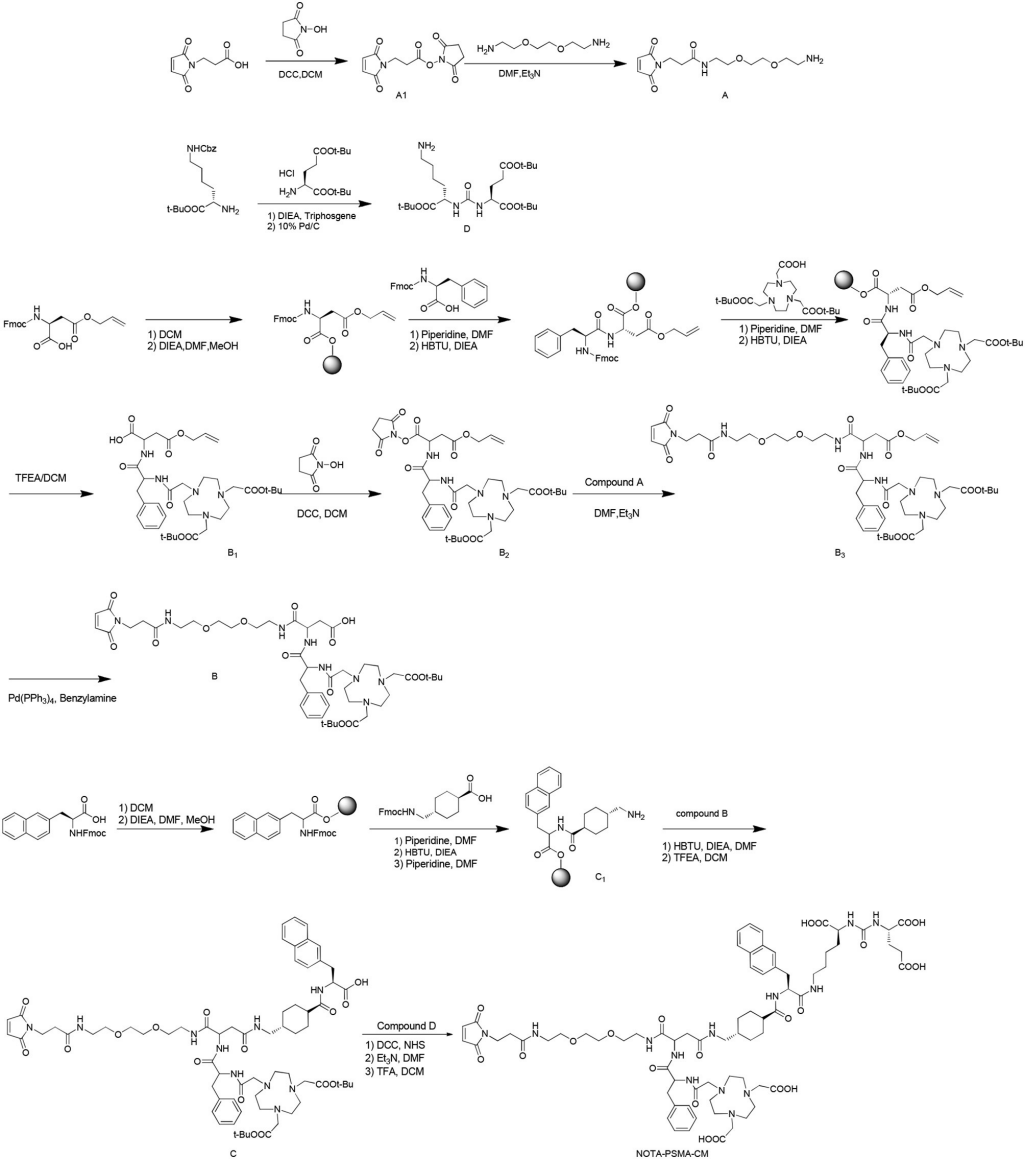
Chemical synthesis of NOTA-PSMA-CM.

NOTA-PSMA-CM was synthesized as Scheme 1.

To a solution of 3-(2,5-dioxo-2,5-dihydro-pyrrol-1-yl)-propionic acid (1 g, 5.92 mmol) in dry dichloromethane (DCM, 100 ml), *N, N*′-dicyclohexylcarbodiimide (DCC, 2.44 g, 11.83 mmol) and *N*-hydroxysuccinimide (NHS, 0.75 g, 6.52 mmol) were added. The mixture was stirred at room temperature overnight. After reaction, the mixture was filtered, and the solution was dried to obtain compound **A1** (1.1 g, yield 70%).

To a solution of 1,8-diamino-3,6-dioxaoctane (0.88 g, 5.92 mmol) in *N, N*-dimethylformamide (DMF, 30 ml), compound **A1** (1.48 g, 5.92 mmol) and triethylamine (TEA, 0.60 g, 5.92 mmol) were added. The mixture was stirred at room temperature for 4 h. After reaction, 30 ml of 10% citric acid solution was added, the mixture was filtered, and the residue was dried to obtain product **A** (0.8 g, yield 45%).

#### Synthesis of Compound B

##### Synthesis of Compound

**B1**2-Chlorotrityl chloride resin (10 g) and DCM (150 ml) were added to a flask, and the mixture was stirred for 30 min. After the solution was filtered, Fmoc-Asp(OAll)-OH (*N*-α-Fmoc-l-aspartic acid β-allyl ester, 1.19 g, 3.02 mmol) and *N, N*-diisopropylethylamine (DIEA, 2.58 g, 20 mmol) in 150 ml of DMF/DCM (1:1) were added, and the mixture was stirred for 30 min and sealed by methanol for 30 min. After removal of the solvent, 150 ml of 20% piperidine solution (in DMF) was added, and the mixture was stirred for 5 min. Then the solvent was removed, 150 ml of 20% piperidine solution (in DMF) was added, and the mixture was stirred for 15 min to remove the Fmoc group. This reaction was monitored as follows: A little of resin was taken out and washed with ethanol (3 × 5 ml), then 50 μl of 25% ninhydrin–alcohol solution and 20% phenolic-alcohol solution and pyridine was added, and the mixture was heated at 105°C for 5 min. The reaction was finished when the color changed to deep blue. After reaction, the resin was washed with DCM (2 × 100 ml), methanol (2 × 100 ml), and DMF (2 × 100 ml). Then *N*-(9-fluorenylmethoxycarbonyl)-l-phenylalanine (1.16 g, 2.99 mmol), *O*-benzotriazole-*N, N, N*′, *N*′-tetramethyl-uronium-hexafluorophosphate (1.14 g, 3.01 mmol), *N, N*-diisopropylethylamine (1.29 g, 9.98 mmol), and DMF (10 ml) were added and reacted for 30 min. This reaction was monitored by ninhydrin as below until colorless. Then the resin was washed by DCM (2 × 100 ml), methanol (2 × 100 ml), and DMF (2 × 100 ml). The reaction was repeated including deprotection, monitoring, washing, condensation, and monitoring to conjugate tri-*tert*-butyl-2,2′,2″-(1,4,7,10-tetraazacyclododecane-1,4,7-triyl)triacetate. After reaction, the protective compound **B1** was obtained by stirring 100 ml of 2,2,2-trifluoroethanol (TFEA):DCM (3:7), filtering, and removing the solvent.

##### Synthesis of Compound **B2**

To a solution of **B1** (1 g, 1.46 mmol) in dry DCM (100 ml), DCC (0.61 g, 2.96 mmol), and NHS (0.19 g, 1.65 mmol) were added, and the mixture was reacted at room temperature overnight. The residue was filtered, and the solution was distilled under vacuum to obtain **B2** (0.86 g, yield 72%).

##### Synthesis of **B3**

Compound **B2** (1.15 g, 1.50 mmol) was dissolved in DMF (30 ml), compound **A** (0.45 g, 1.50 mmol) and triethylamine (0.45 g, 4.50 mmol) were added, and the mixture was reacted at room temperature for 4 h. After reaction, 30 ml of 10% citric acid solution was added, the mixture was filtered, and the residue was dried to obtain product **B3** (1.15 g, yield 77%).

##### Synthesis of **B**

To a solution of **B3** (0.96 g, 0.99 mmol) in dry DCM (100 ml), tetrakis(triphenylphosphine)palladium (58 mg, 0.05 mmol) and benzylamine (54 mg, 0.50 mmol) were added and stirred at 25°C overnight. After reaction, the mixture was filtered, the solvent was removed, and the residue was purified by silica gel flash column chromatography (methanol/DCM/acetic acid 0.5%, 0%−10%, vol/vol) to obtain product **B** (0.44 g, yield 46%).

#### Synthesis of Compound C

Compound **C** was synthesized as compound **B1** with the reagents of Fmoc-2-Nal-OH (2-({[(9*H*-fluoren-9-yl)methoxy]carbonyl}amino)-3-(naphthalen-2-yl), Fmoc-tranexamic acid {(1r,4r)-4-[({[(9H-fluoren-9-yl)methoxy]carbonyl}amino)methyl]cyclohexane-1-carboxylic acid} and compound **B** sequentially.

#### Synthesis of Compound D

Triphosgene (0.29 g, 0.98 mmol) was dissolved in anhydrous DCM (20 ml) at −10°C. To the solution, a mixture of (*S*)-*tert*-butyl 2-amino-6-{[(benzyloxy)carbonyl]amino}hexanoate hydrochloride (1 g, 2.68 mmol) and *N, N*-diisopropylethylamine (0.69 g, 5.34 mmol) in anhydrous DCM (10 ml) was added in 5 min. The reaction mixture was stirred at −10°C for 2 h under nitrogen, and then a solution of (*S*)-di-*tert*-butyl-2-aminopentanedioate hydrochloride (0.79 g, 2.68 mmol) and *N, N*-diisopropylethylamine (0.69 g, 5.34 mmol) in anhydrous DCM (10 ml) was added over 30 min. The reaction was stirred for another 5 h. After reaction, the solvent was removed by vacuum distillation, and 20 ml of ethyl acetate was added and extracted by saturated salt water (3 × 15 ml). The organic phase was dried by anhydrous MgSO_4_ and distilled under vacuum to obtain the crude product. The crude product was dissolved in ethanol (30 ml), 10% Pd/C was added, and the mixture was stirred under hydrogen at room temperature overnight. Then Pd/C was removed, and the solvent was removed to obtained precursor **D** (0.67 g, yield 51% for two steps).

#### Synthesis of NOTA-PSMA-CM

To a solution of compound **C** (1.30 g, 1.03 mmol) in DCM (20 ml), DCC (0.42 g, 2.04 mmol) and NHS (0.13 g, 1.13 mmol) were added under the temperature of ≤ 5°C, and the mixture was stirred at room temperature overnight. After reaction, the mixture was filtered, the solvent was removed, and the residue was dissolved in DMF (20 ml). To the solution, compound **D** (0.62 g, 1.27 mmol) and Et_3_N (0.31 g, 3.09 mmol) were added, and the mixture was stirred at ≤ 5°C for 30 min and then at room temperature overnight. After reaction, the solvent was removed under vacuum, the residue was dissolved in a mixture of DCM (10 ml) and TFA (10 ml), the reaction mixture was stirred at room temperature overnight, the solvent was evaporated, and the residue was purified by HPLC to obtain product NOTA-PSMA-CM (0.38 g, yield 25% for three steps), with a purity of 98.32%. ^1^H NMR (600 MHz, DMSO) δ 8.03 (t, *J* = 5.5 Hz, 1H), 7.97–7.95 (m, 2H), 7.83 (d, *J* = 7.9 Hz, 1H), 7.78 (t, *J* = 7.6 Hz, 2H), 7.72 (brs, 1H), 7.68 (s, 1H), 7.48–7.37 (m, 3H), 7.22–7.12 (m, 6H), 7.06 (brs, 1H), 7.00 (s, 2H), 6.55 (s, 1H), 6.33 (d, *J* = 8.2 Hz, 1H), 6.29 (d, *J* = 8.3 Hz, 1H), 4.57–4.52 (m, 2H), 4.13–4.09 (m, 1H), 4.04–4.00 (m, 2H), 3.63–3.57 (m, 2H), 3.51–3.47 (m, 4H), 3.42–3.35 (m, 6H), 3.22–2.99 (m, 10H), 3.00–2.87 (m, 8H), 2.85–2.66 (m, 6H), 2.49–2.44 (m, 2H), 2.36–2.31 (m, 2H), 2.29–2.20 (m, 2H), 2.12–1.87 (m, 4H), 1.76–1.54 (m, 6H), 1.51–1.44 (m, 2H), 1.35–1.30 (m, 2H), 1.24–1.18 (m, 6H), 1.12–1.05 (m, 1H), 0.82–0.72 (m, 2H). ^13^C NMR (600 MHz, DMSO) δ 175.14, 174.55, 174.17, 173.72, 170.97, 170.91, 170.74, 170.53, 169.52, 169.39, 157.86, 157.67, 157.29, 138.21, 135.69, 134.55, 132.87, 131.76, 129.64, 129.28, 128.11, 127.87, 127.43, 127.39, 127.29, 127.24, 126.10, 125.90, 125.31, 69.49, 68.99, 68.63, 53.70, 52.26, 51.65, 50.57, 48.80, 44.78, 43.67, 40.05, 38.70, 38.47, 38.36, 38.17, 37.38, 37.00, 35.08, 34.06, 33.93, 31.68, 29.90, 29.66, 29.49, 28.69, 28.56, 28.37, 27.53, 26.58, 26.53, 25.07, 22.55, 22.06. ESI-MS: 1,485.7 ([M+H]^+^, C_71_H_98_N_13_O_22_, calculated 1,485.6), 743.1 ([M+2H]^2+^/2, calculated 743.3). ^1^H NMR, ^13^C NMR, and MS spectra were shown in Supplementary Figures 1–3.

### Radiosynthesis

**Scheme 2:**
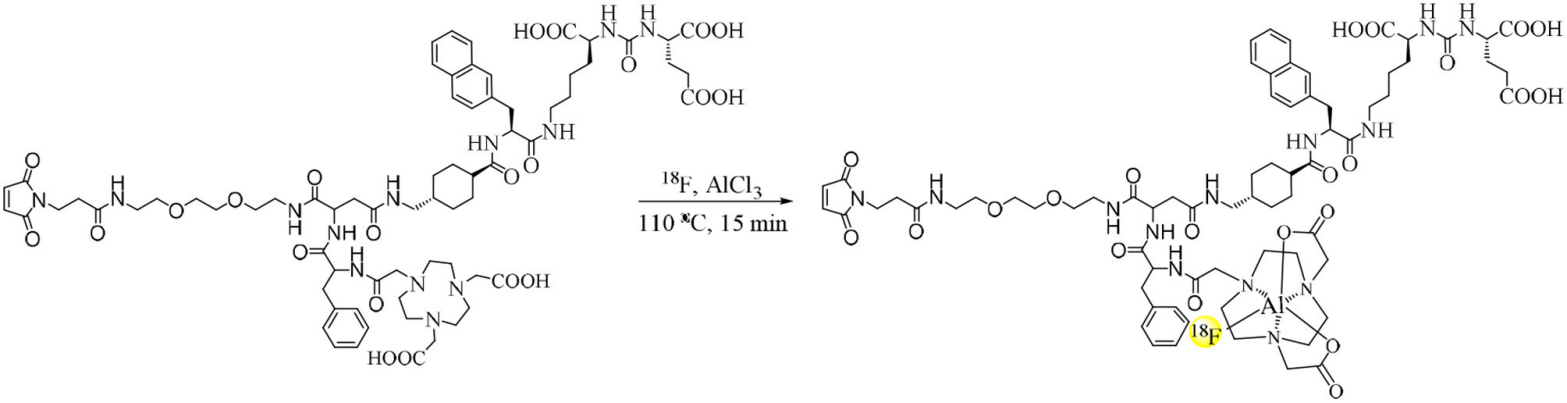
Radiosynthesis of Al^18^F-PSMA-CM.

No-carrier-loaded ^18^F^−^ was eluted from a QMA cartridge by 0.4 ml of saline. As shown in Scheme 2, 11 μl of KHP (0.5 mol/L), 100 μl of ^18^F^−^ in saline (1.1–1.8 GBq), and 6 μl of AlCl_3_ (2 mmol/L) were added to a tube and reacted at room temperature for 5 min. The solution was added with 5 μl of NOTA-PSMA-CM (4 mmol/L) and was reacted at 100°C for 15 min. After reaction, the solution was diluted with 4 ml of H_2_O and loaded on a C18 Light cartridge. Then the cartridge was washed with 5 ml H_2_O, and Al^18^F-PSMA-CM was obtained by eluting the cartridge with 0.6 ml of 80% ethanol and 5 ml of saline.

### Quality Control

The pH of the solution was determined. The radiochemical purity of Al^18^F-PSMA-CM was analyzed by radio-HPLC. Radio-HPLC was conducted on the Agilent 1200 system equipped with a UV detector (220 nm) with a C18 cartridge (ZORBAX 300SB-C18, 4.6 × 250 mm, 5 μm; Agilent, USA). The mobile phases were H_2_O (1% TFA, A) and acetonitrile (1% TFA, B, 15–60% of B in 15 min) with a flow of 1 ml/min.

### Stability

The *in vitro* stability of Al^18^F-PSMA-CM in saline and 5% HSA was detected. Al^18^F-PSMA-CM (3.7 MBq) of 20 μl was added to 1 ml of saline and incubated at 37°C, and the chemical purity was analyzed at 5, 30, 60, and 120 min. For the stability in 5% HSA, 20 μL of Al^18^F-PSMA-CM (3.7 MBq) was added to 500 μl of 5% HSA and incubated at 37°C, after 5, 30, 60, 120, and 240 min; 100 μl of the solution was taken out, precipitated with 100 μl of ethanol, and filtered; and the solvent was passed through a 0.22-μm filter and analyzed by radio-HPLC.

### Partition Coefficient

Ten microliters of Al^18^F-PSMA-CM (1.8 MBq), 1 ml of PBS (pH 7.4), and 1 ml of *n*-octanol were mixed in a tube, and then the mixture was vortexed for 3 min and centrifuged (1,000 r/min × 5 min). Five samples in each phase were taken out and measured for the radioactivity. The experiment was repeated three times. The result was presented as logP ± SD, where P = counts of *n*-octanol/counts of PBS.

### Binding Affinity

Ten microliters of Al^18^F-PSMA-CM (1.8 MBq) was added to 0.1 ml of 20% HSA (*n* = 5) and incubated at 37°C for 1 h. Then the protein was precipitated by 0.2 ml of ethanol, washed by saline (3 × 0.3 ml), and measured by a γ-counter for radioactivity. As standard, 10 μl of Al^18^F-PSMA-CM (1.8 MBq) was diluted with 1 ml saline, and three samples of 10 μl were taken out and measured for radioactivity. The result was presented as the percentage of added dose (%). The same study of Al^18^F-PSMA-BCH was performed (Liu et al., 2019).

The Kd values of Al^18^F-PSMA-CM to HSA were tested as follows. A 96-well polystyrene Stripwell™ microplate was coated with 50 μl of 20% HSA per well and then stored at 4°C overnight. The solution was removed, and the microplate was washed three times with PBS. Then 50 μl of 5% powdered milk diluted with PBS was added to each well of the microplate, and the microplate was stored at room temperature for 2 h. After removal of the solution, the microplate was washed five times with PBS and stored at 4°C. Different concentrations (0.01–50 mCi/ml) of Al^18^F-PSMA-CM were added to the wells of the microplate (50 μl per well, four wells per group), and the microplate was stored at 37°C for 2 h. Then the solution was removed, and the microplate was washed three times with PBS. Each well was separated, and the radioactivity of the well was measured. With a similar protocol, the Kd value of Al^18^F-PSMA-BCH to HSA was tested. For the Kd value of Al^18^F-PSMA-CM to PSMA, the microplate was coated with 2 μg/ml of PSMA, and the value was tested with a similar protocol.

### Cell Culture and Tumor Models

The 22Rv1 (PSMA+) cell line was provided by the Stem Cell Bank, Chinese Academy of Sciences. 22Rv1 cells were cultured in RPMI-1640 medium (Gibco) supplemented with 10% fetal bovine serum (Gibco) and 1% penicillin–streptomycin solution. Cells were incubated in a humidified incubator at 37°C with 5% CO_2_. Into the right axilla of male BALB/c nude mice were injected 0.1 mL 22Rv1 cells (2 × 10^7^ cells/ml). When the tumors grew up to 0.5–1 cm in diameter, the mice underwent biodistribution and micro-PET imaging studies. All animal studies were performed according to a protocol approved by the Peking University Cancer Hospital Animal Care and Use Committee.

### Cell Uptake

22Rv1 cells were placed in 24-well-plates (2 × 10^5^ cells per well) 24 h before the study, the medium was removed, and 0.5 ml of fresh medium was added 2 h before the study. Al^18^F-PSMA-CM (37 kBq) of 10 μl was added to each well. Five, 30, 60, and 120 min later, the medium was removed, and the cells were washed twice with cold PBS (2 × 1 ml) and lysed by cold NaOH (0.5 ml, 1 mol/L). The radioactivity was measured by a γ-counter. For blocking, 1 μg of the PSMA inhibitor ZJ-43 [(*S*)-2-{3-[(*S*)-1-carboxy-3-methylbutyl]ureido}, pentanedioic acid] was added to each well.

### Pharmacokinetics in Blood

Healthy BALB/c male mice (*n* = 5) were intravenously injected with 200 μl of Al^18^F-PSMA-CM (3.7 MBq). Blood was collected from the ophthalmic artery at 2, 5, 10, 15, 30, 45, 60, 90, 120, 180, and 240 min p.i. Then the blood was weighted and measured for radioactivity by a γ-counter. The results were expressed as the percentage of injected dose per gram (%ID/g). For comparison, the pharmacokinetics of Al^18^F-PSMA-BCH was studied.

A two-compartment model was used to describe the blood pharmacokinetics of Al^18^F-PSMA-CM and Al^18^F-PSMA-BCH, whose corresponding equations were obtained by fitting the percentage of the injected dose per gram (ID%/g) versus time to the following equation:

$$\begin{array}{l} C_{t}=Ae^{-\text{α}t}+Be^{-\text{β}t} \end{array}$$

A and B are the relevant constants for the model, and α and β are two rate constants.

### Biodistribution

Al^18^F-PSMA-CM (0.74 MBq) of 200 μl was intravenously injected into BALB/c nude male mice bearing 22Rv1 via the tail vein. Mice were sacrificed (four mice per group) by cervical dislocation at 1, 2, and 4 h p.i. For blocking, mice were co-injected with ZJ-43 (50 μg) and sacrificed at 2 h p.i. Heart, liver, lung, kidneys, spleen, stomach, bone, muscle, brain, blood, intestines, and tumor were collected, weighted, and measured for radioactivity by a γ-counter. As a standard, 10 samples of 1% injected dose were taken out and measured for radioactivity. The biodistribution of Al^18^F-PSMA-BCH in mice bearing 22Rv1 tumor at 4 h p.i. was studied with a similar method. The results were expressed as the percentage of injected dose per gram (%ID/g).

### Micro-PET Imaging

BALB/c nude mice bearing 22Rv1 xenograft tumor were intravenously injected with 200 μl of Al^18^F-PSMA-CM (7.48 MBq) via the tail vein. For blocking, 50 μg of ZJ-43 was co-injected. The mice were anesthetized with 3% (v/v) isoflurane at 1, 2, and 4 h p.i., and then micro-PET imaging was performed with continuous 1.5% (v/v) isoflurane.

Imaging was performed on the Super Argus PET system (Sedecal, Spain) acquired with an 80-mm-diameter transaxial FOV and OSEM 3D reconstruction algorithms with attenuation and random corrections. Finally, the images were displayed by MMWKS Super Argus. The millicounts per second and SUV values of regions of interest (ROIs) over the tumor, kidney, liver, heart, and muscle were collected.

### Human Organ Radiation Dosimetry Estimates

With the biodistribution data of Al^18^F-PSMA-CM in mice bearing 22Rv1, human organ dosimetry was estimated using the OLINDA/EXM software package (Green et al., 2017). The effective dose was calculated as the sum of the absorbed dose plus the tissue weighting factors of each organ.

### Statistical Analysis

The data were analyzed by GraphPad Prism 5 software and reported as mean ± SD. A *P* < 0.05 was considered statistically significant.

## Results

### Radiosynthesis and Quality Control

**Figure 1:**
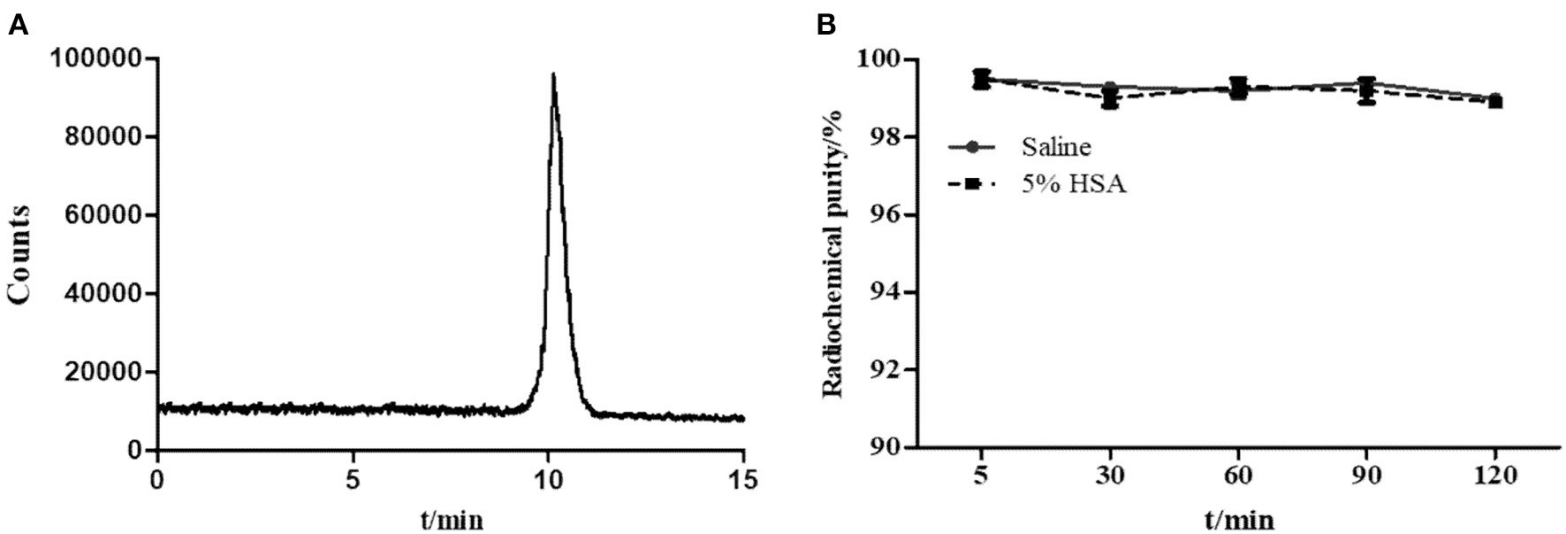
**(A)** Radio-HPLC pattern of Al^18^F-PSMA-CM. **(B)** The radiochemical purity of Al^18^F-PSMA-CM in saline and 5% HSA at 37°C for 4 h.

Al^18^F-PSMA-CM was prepared and characterized by radio-HPLC for radiochemical purity. HPLC showed a retention time (t_R_) of 10.14 min (Figure 1A), while that of ^18^F^−^ was 3.0 min. The non-decayed radiochemical yield of Al^18^F-PSMA-CM was calculated as 34.2 ± 5.2% with a radiochemical purity of >99% and a specific activity of 15.2 ± 2.9 GBq/μmol. The pH value of the injection was 6.7–7.4 with an ethanol concentration of <10%.

### Stability

The *in vitro* stability of Al^18^F-PSMA-CM was analyzed by radio-HPLC. After incubation in saline and in 5% HSA for 4 h, the radiochemical purity was over 98% (Figure 1B).

### Partition Coefficient

The partition coefficient of Al^18^F-PSMA-CM was measured in a PBS–octanol system with a logP value of −2.25 ± 0.05, which indicated that Al^18^F-PSMA-CM was hydrophilic but more lipophilic than the reported Al^18^F-PSMA-BCH without MPA moiety (logP = −2.87 ± 0.01) (Liu et al., 2019).

### Cell Uptake

**Figure 2:**
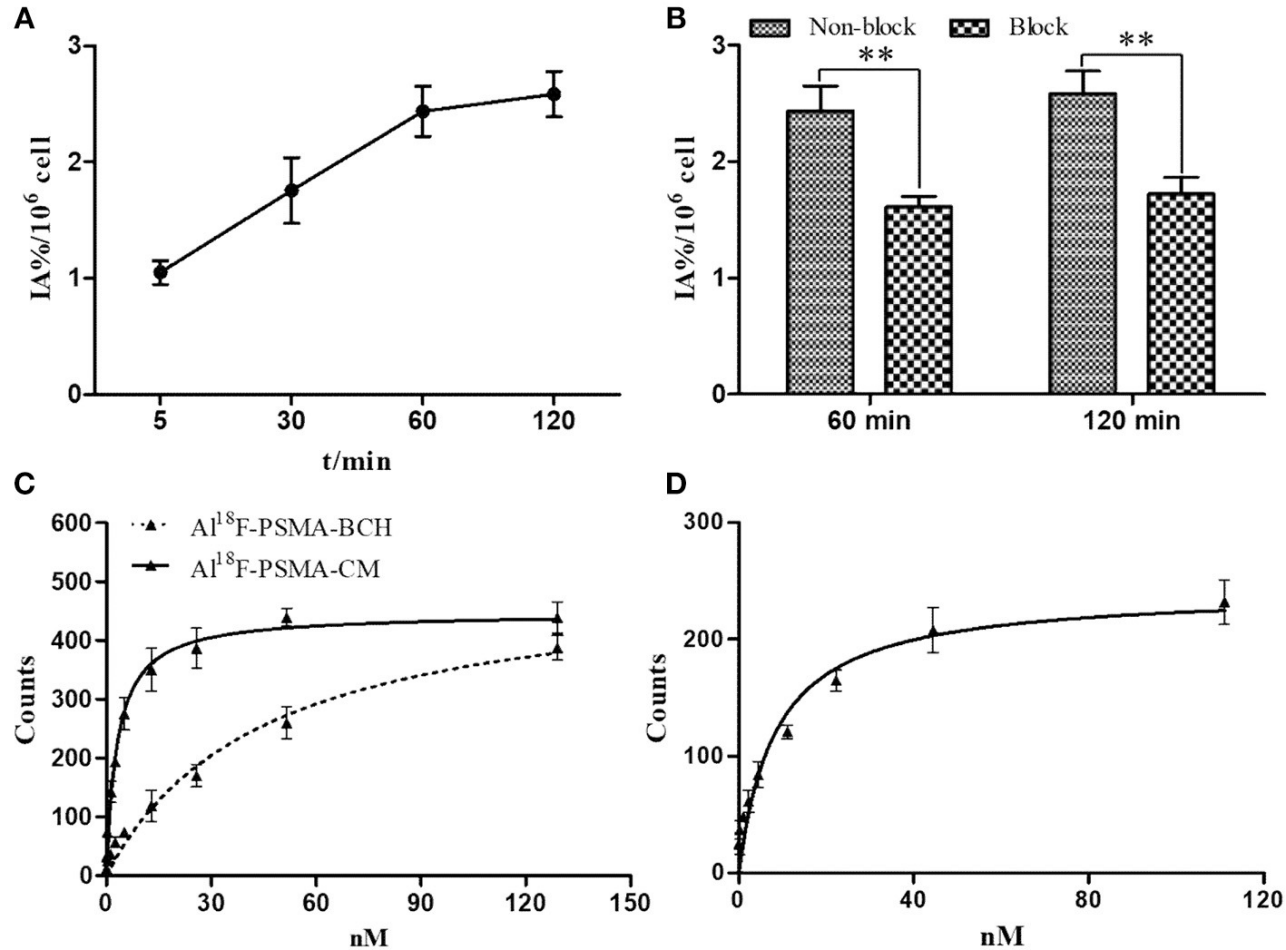
**(A)** Cell uptake of Al^18^F-PSMA-CM in 22Rv1 cells with time; **(B)** cell uptake of Al^18^F-PSMA-CM in 22Rv1 cells with or without ZJ-43 (1 μg per well); **(C)** binding affinity of Al^18^F-PSMA-CM and Al^18^F-PSMA-BCH to HSA; **(D)** binding affinity of Al^18^F-PSMA-CM to PSMA.

An *in vitro* cell uptake study of Al^18^F-PSMA-CM was performed on the 22Rv1 cell line, which slightly expresses PSMA (Gorges et al., 2016). The uptake in 22Rv1 cells was increased over time, and the highest uptake was 2.58 ± 0.19 IA%/10^6^ cells at 2 h, while the uptake was decreased when excess ZJ-43, a PSMA inhibitor, was added. The uptake was blocked by 34.0% (2.44–1.61 IA%/10^6^ cells) and 33.3% (2.58–1.72 IA%/10^6^ cells) at 1 and 2 h, respectively (Figures 2A,B).

### Binding Affinity

In order to study the binding affinity of Al^18^F-PSMA-CM to albumin, an HSA binding study was performed. The binding rates of Al^18^F-PSMA-CM and Al^18^F-PSMA-BCH to HSA were 57.2 and 32.1%, respectively (Liu et al., 2019). The Kd values of Al^18^F-PSMA-CM and Al^18^F-PSMA-BCH were 3.08 ± 0.45 and 45.18 ± 1.20 nM, respectively. The Kd value of Al^18^F-PSMA-CM to PSMA was 8.46 ± 0.24 nM (Figures 2C,D).

### Pharmacokinetics

**Figure 3:**
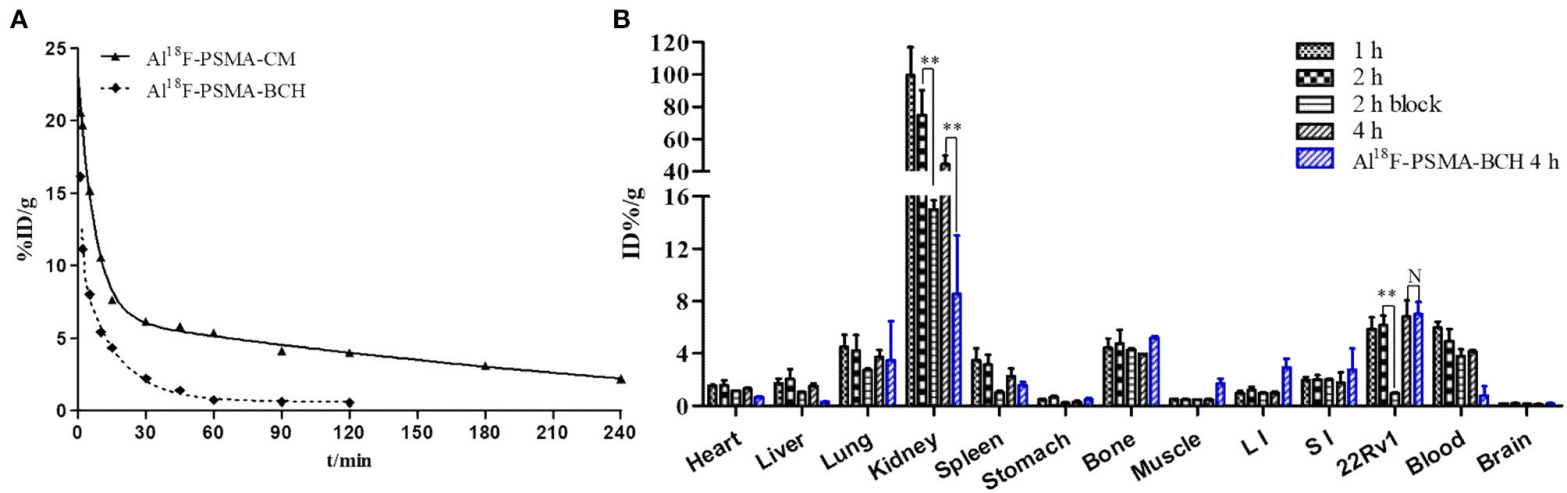
**(A)** Pharmacokinetics of Al^18^F-PSMA-CM and Al^18^F-PSMA-BCH in BALB/c male mice. **(B)** Biodistribution of Al^18^F-PSMA-CM and Al^18^F-PSMA-BCH in BALB/c nude male mice bearing 22Rv1 tumor with or without 50 μg of ZJ-43 (block) (*n* = 4). **: *P* < 0.01, N: *P* > 0.5.

The equation for Al^18^F-PSMA-CM was C_*t*_ = 16.846e^−0.136t^ + 9.004e^−0.003t^ with a half-life of 5.11 min for the distribution phase and 210.96 min for the elimination phase. The equation for Al^18^F-PSMA-BCH was C_*t*_ = 20.600e^−1.174t^ + 9.757e^−0.064t^ with a half-life of 0.59 min for the distribution phase and 10.81 min for the elimination phase (Liu et al., 2019) (Figure 3A).

### Biodistribution

Mice bearing 22Rv1 xenograft tumors were intravenously injected with 200 μl Al^18^F-PSMA-CM (185 kBq) and sacrificed at 1, 2, and 4 h p.i. Compared with Al^18^F-PSMA-BCH, Al^18^F-PSMA-CM showed prolonged circulation *in vivo* and higher uptake in organs (Figure 3B). It was highly accumulated in kidneys with uptake values of 99.46 ± 17.28 ID%/g at 1 h p.i. and 44.44 ± 5.43 ID%/g at 4 h p.i. The clearance in the blood was slow with uptake values of 5.99 ± 0.44 ID%/g at 1 h p.i. and 4.16 ± 0.12 ID%/g at 4 h p.i. 22Rv1 tumor showed a slightly increased uptake of 6.83 ± 1.16 ID%/g at 4 h p.i. As with most PSMA probes, the uptake values of Al^18^F-PSMA-CM in kidneys (−79.9%, 74.76–14.99 ID%/g) and 22Rv1 tumor (−84.3%, 6.16–0.97 ID%/g) at 2 h p.i. were decreased when 50 μg of ZJ-43 was co-injected. Compared with that of Al^18^F-PSMA-BCH, the uptake values of Al^18^F-PSMA-CM in the blood, heart, liver, and spleen were higher, while those in 22Rv1 tumor were 6.83 ± 1.16 and 7.01 ± 1.17 ID%/g (*P* = 0.54) for Al^18^F-PSMA-CM and Al^18^F-PSMA-BCH, respectively.

### Micro-PET Imaging

**Figure 4:**
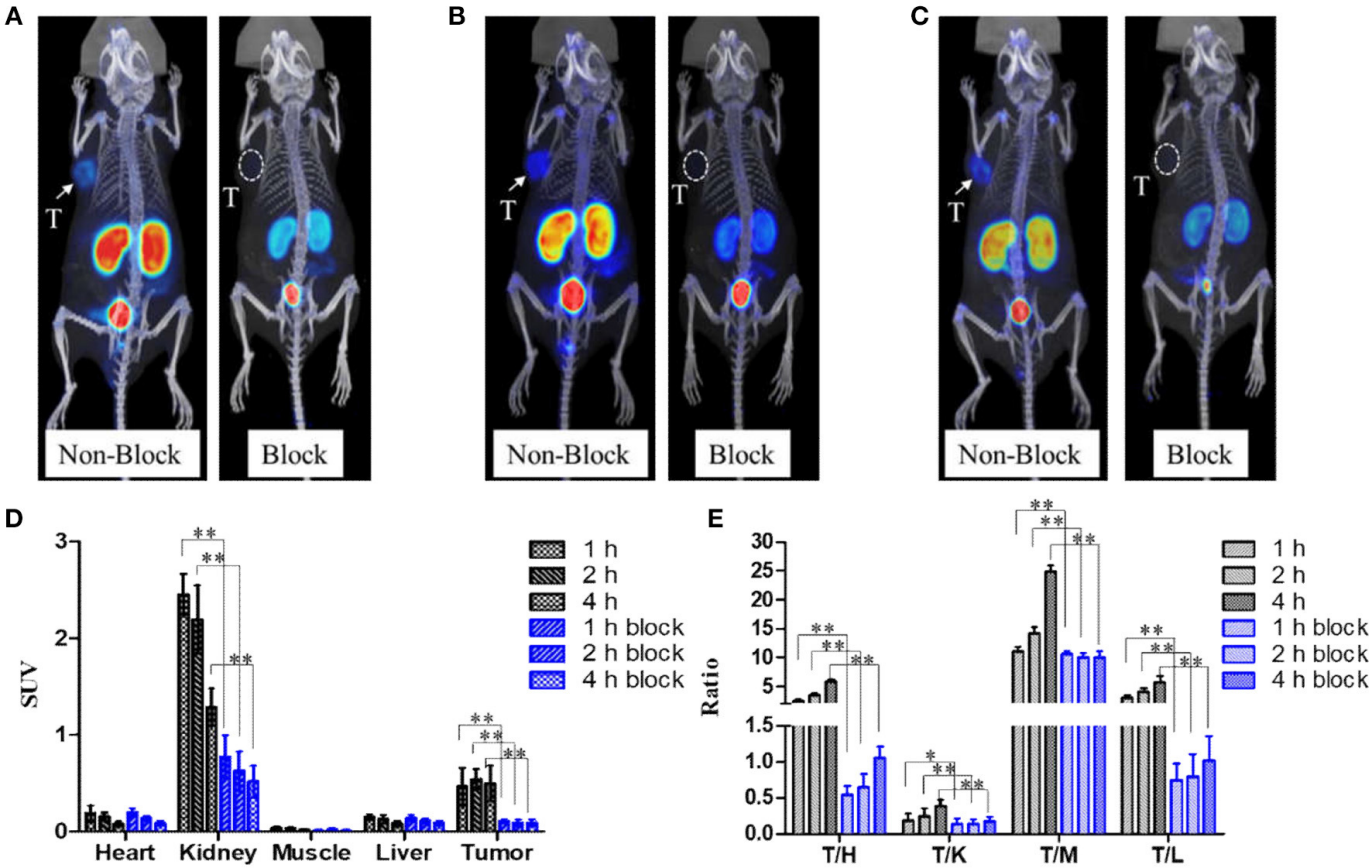
Micro-PET images of Al^18^F-PSMA-CM in mice bearing 22Rv1 with (block) or without (non-block) 50 μg of ZJ-43; white arrows and white dotted circles indicate 22Rv1 tumors. **(A)** Images at 1 h p.i. **(B)** Images at 2 h p.i. **(C)** Images at 4 h p.i. **(D)** Uptake of tumor and other organs at 1, 2, and 4 h p.i. obtained by ROI. **(E)** 22Rv1-to-background ratios at 1, 2, and 4 h p.i. *: P < 0.05, **: P < 0.01, T/H, tumor/heart; T/K, tumor/kidney; T/M, tumor/muscle; T/L, tumor/liver.

Mice bearing 22Rv1 tumor underwent micro-PET imaging at 1, 2, and 4 h p.i. (Figure 4). Kidneys, bladder, and 22Rv1 tumor were clearly observed; the uptake in kidneys decreased over time, while the accumulation in 22Rv1 tumor increased between 1 and 2 h p.i. and was maintained between 2 and 4 h p.i. (*P* > 0.05), which coincides with the biodistribution results. Because of the clearance in non-target organs, the tumor-to-background ratios were increased with time. When co-injected with 50 μg of ZJ-43, 22Rv1 tumor was invisible and the uptake in kidneys was decreased, while the uptake values in the liver, heart, and muscle were similar to that of Al^18^F-PSMA-CM without ZJ-43. Because of the resistance of radioactivity in tumor and clearance in non-target organs of mice without ZJ-43, the tumor-to-non-target organ ratios were increased at 4 h p.i., while the ratios in mice with ZJ-43 were increased slightly or kept.

### Human Organ Radiation Dosimetry Estimates

Human organ radiation dosimetry was estimated during the biodistribution of Al^18^F-PSMA-CM in mice bearing 22Rv1 with the OLINDA/EXM 2.0 software package. As shown in Table 1, kidneys are the most critical organ with an absorbed dose of 0.141 mGy/MBq, and the salivary glands are the second most critical organs with an absorbed dose of 0.0613 mGy/MBq. The effective dose is calculated as 0.019 mSv/MBq.

**Table 1:** Human organ radiation dosimetry estimates for Al^18^F-PSMA-CM.

**Target organ**	**Absorbed dose (mGy/MBq)**
Adrenals	1.99E−02
Brain	1.09E−03
Esophagus	4.66E−03
Eyes	3.56E−03
Gallbladder wall	6.22E−03
Left colon	7.80E−02
Small intestine	6.89E−02
Stomach wall	6.26E−02
Right colon	6.56E−02
Rectum	5.61E−02
Heart wall	4.68E−02
Kidneys	1.39E+01
Liver	4.68E−02
Lungs	2.98E−02
Pancreas	6.59E−02
Prostate	5.77E−02
Salivary glands	4.20E−02
Red marrow	4.72E−02
Osteogenic cells	4.22E−02
Spleen	1.09E−01
Testes	4.32E−02
Thymus	4.28E−02
Thyroid	4.36E−02
Urinary bladder wall	5.35E−02
Total body	5.17E−02
Effective dose (mSv/MBq)	0.0589

## Discussion

^177^Lu/^225^Ac-PSMA-based PRLT demonstrated favorable prognosis and was thought as a potential therapeutic option for mCRPC patients. The most studied PRLT radiotracers were fast cleared out from the body due to their short half-life, which lead to more frequent injection or higher dose of radioactivity to reach therapeutic level. Many studies have reported different strategies to prolong the half-life of tracers. HSA is an ideal versatile carrier for prolonging the profile of fast-clearance drugs as it is the most abundant circulating protein in plasma. In this study, MPA moiety was used as a platform for targeting albumin and conjugating with the PSMA tracer. In order to verify its ability of prolonging the half-life in the blood and evaluating the influence of MPA on the specificity to PSMA, we chemically synthesized a NODA-conjugated MPA-PSMA precursor named PSMA-CM and radiolabeled it with ^18^F to obtain Al^18^F-PSMA-CM with the strategy of aluminum fluoride.

Al^18^F-PSMA-CM was prepared with high radiochemical purity, yield, specific activity, and stability in saline and in 5% HSA for 4 h at 37°C. The introduction of lipophilic MPA moiety and phenylalanine increased the lipophilicity of Al^18^F-PSMA-CM, but it was still hydrophilic. The conjugation of MPA had little influence on the physicochemical properties. Al^18^F-PSMA-CM was qualified and can be used for further biological studies.

Compared with Al^18^F-PSMA-BCH, a tracer without MPA moiety, Al^18^F-PSMA-CM showed higher binding affinity to albumin but lower binding affinity to PSMA. The MPA moiety extended the circulation of radiotracers in the blood.

Though 22Rv1 cells slightly express PSMA, it is easy for culture and establishment of tumor models, and it was used to evaluate the specificity of Al^18^F-PSMA-CM to PSMA in this study. The uptake of Al^18^F-PSMA-CM in 22Rv1 cells was increased over 2 h, and the uptake can be blocked by co-incubation with excess ZJ-43, a PSMA inhibitor, at 1 and 2 h.

In mice bearing 22Rv1 tumor, Al^18^F-PSMA-CM was mainly accumulated in the kidneys and bladder; this was because Al^18^F-PSMA-CM was excreted mostly by the urinary system and the kidneys express PSMA, which coincides with the fact that uptake in the kidneys can be blocked by ZJ-43. The uptake in 22Rv1 tumor was increased within 4 h p.i., indicating that the extended circulation *in vivo* may have a positive effect on the accumulation of radioactivity in PSMA-expressed tumors. Compared with the biodistribution of Al^18^F-PSMA-BCH, the uptake of Al^18^F-PSMA-CM in tumor was lower without significant differences. The lower binding affinity of Al^18^F-PSMA-CM to PSMA displayed a similar tumor uptake to that of Al^18^F-PSMA-BCH, indicating that the existence of MPA increased the uptake of radiotracers in the tumor by extending the circulation in the blood. The radiation dosimetry estimates indicated that the combination of MPA increased the effective dose of Al^18^F-PSMA-CM.

In this study, MPA combined with a PSMA tracer, Al^18^F-PSMA-CM, was proven to have high PSMA specificity, longer half-life in the blood, and high tumor uptake, indicating that the introduction of MPA to PSMA-targeted tracers is a good strategy to extend the half-life of tracers. Designing and optimizing a DOTA-conjugated MPA-PSMA precursor and radiolabeling with ^177^Lu or ^225^Ac are expected to be used in PRLT of PCa.

## Conclusion

An MPA-conjugated PSMA tracer was prepared and radiolabeled with ^18^F to obtain Al^18^F-PSMA-CM; it showed good physicochemical and biological properties. This study initially proved the efficiency of MPA for prolonging the circulation of tracers in the blood and the potential for increasing the accumulation of radioactivity in PSMA-expressing tumors. Though the introduction of MPA increased the uptake in kidneys, it is a promising approach for designing PRLT radiopharmaceuticals.

## Data Availability Statement

The authors acknowledge that the data presented in this study must be deposited and made publicly available in an acceptable repository, prior to publication. Frontiers cannot accept a article that does not adhere to our open data policies.

## Ethics Statement

The animal study was reviewed and approved by Ethics Committee of Beijing Cancer Hospital.

## Author Contributions

TL designed this study, performed most of the experiments, and wrote this article. CL was involved in establishing the tumor models, in the micro-PET imaging, and in the corresponding data analysis. YR helped with the micro-PET imaging. XG helped with the biodistribution study. JJ helped with the pharmacokinetics study. QX helped with the offering of ^18^F^−^. LX helped with the cell uptake study. FW helped with the estimates of radiation dosimetry. HZ and ZY helped design this study and reviewed this article. All authors contributed to the article and approved the submitted version.

## Conflict of Interest

The authors declare that the research was conducted in the absence of any commercial or financial relationships that could be construed as a potential conflict of interest.
