# Supplementary material for: Development of an Albumin-Based PSMA Probe With Prolonged Half-Life
Source: Front Mol Biosci. 2020 Dec 17;7:585024. doi: 10.3389/fmolb.2020.585024 (PMC7773938; doi:10.3389/fmolb.2020.585024)
Supplement: Supplementary file 1 [file Data_Sheet_1.PDF]

Supporting information of

Development of an albumin-based PSMA probe with prolonged half-life

Teli Liu[1,#], Chen Liu[1,#], Yanan Ren[1,2], Xiaoyi Guo[1], Jinquan Jiang[1], Qing Xie[1], Lei Xia[1], Feng Wang[1], Hua Zhu[1,*], Zhi Yang[1,*]

[1]Key Laboratory of Carcinogenesis and Translational Research (Ministry of Education/Beijing), Department of Nuclear Medicine, Peking University Cancer Hospital & Institute, Beijing, 100142, China

[2]Guizhou University School of Medicine, Guizhou University, Guiyang 550025, China

[#] They contributed equally to this work

*** Correspondence:**
Hua Zhu
zhuhuananjing@163.com

Zhi Yang
pekyz@163.com

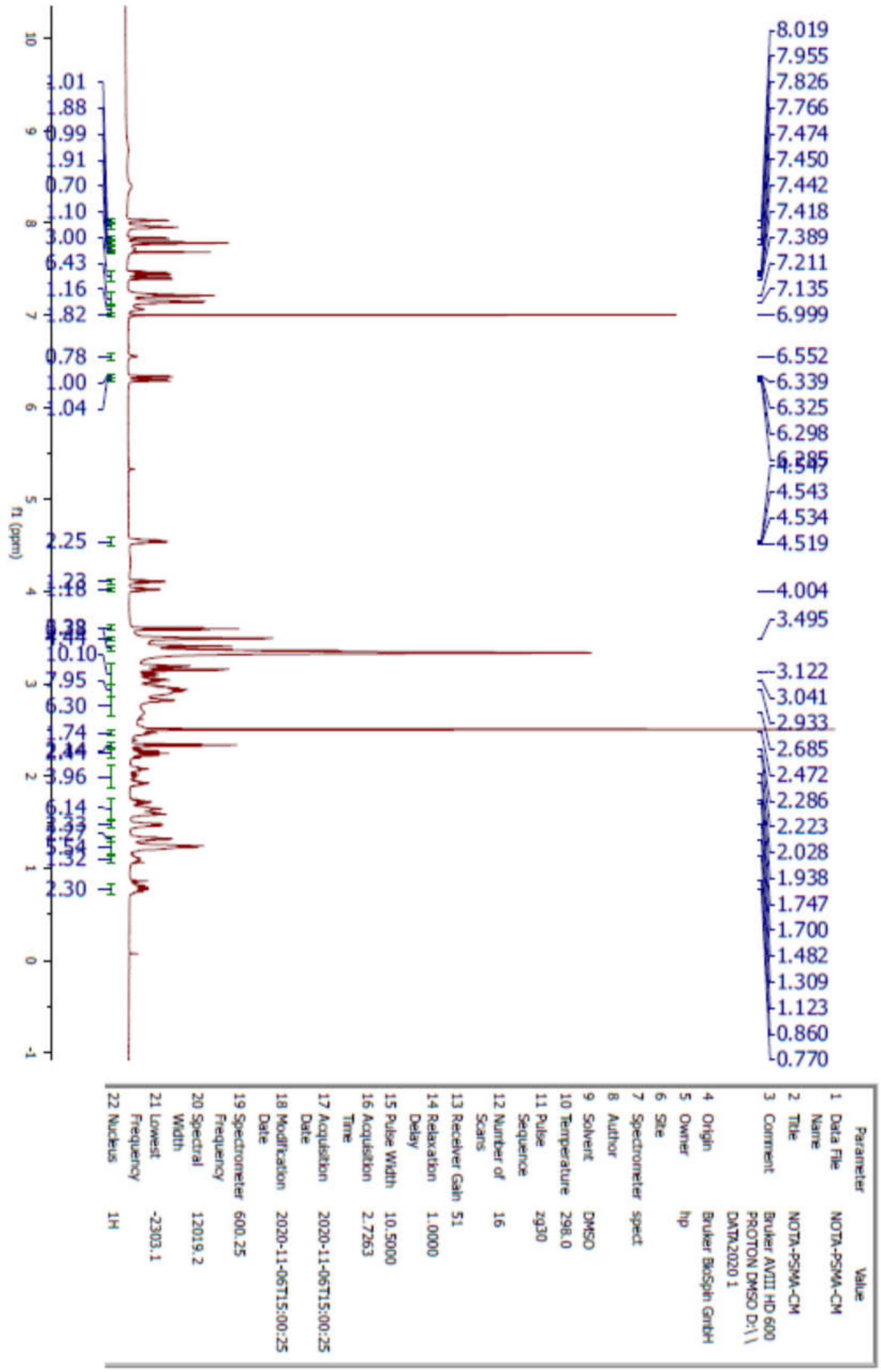

Figure S1. ^{1}H NMR spectrum of NOTA-PSMA-CM

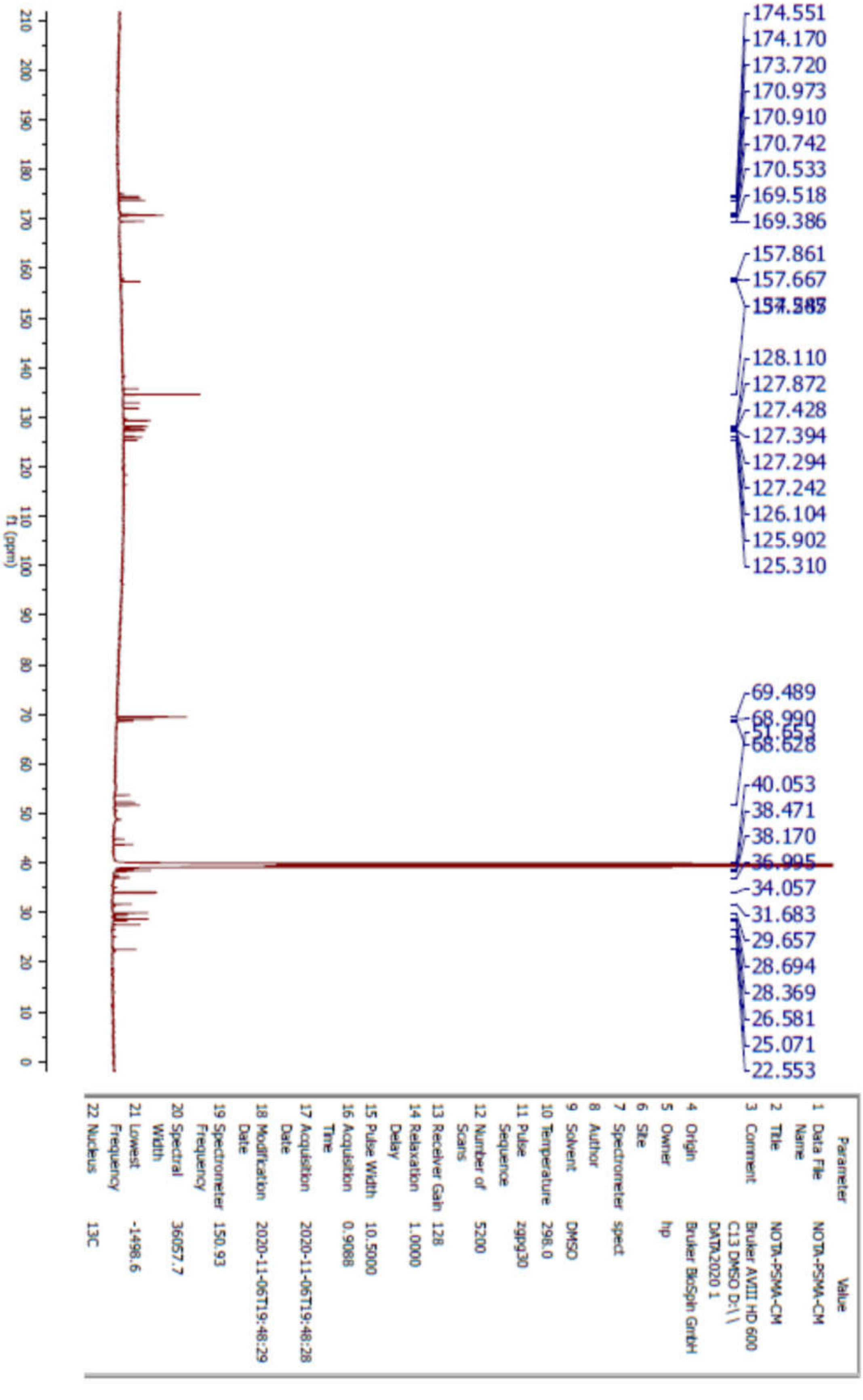

Figure S2. ^{13}C NMR spectrum of NOTA-PSMA-CM

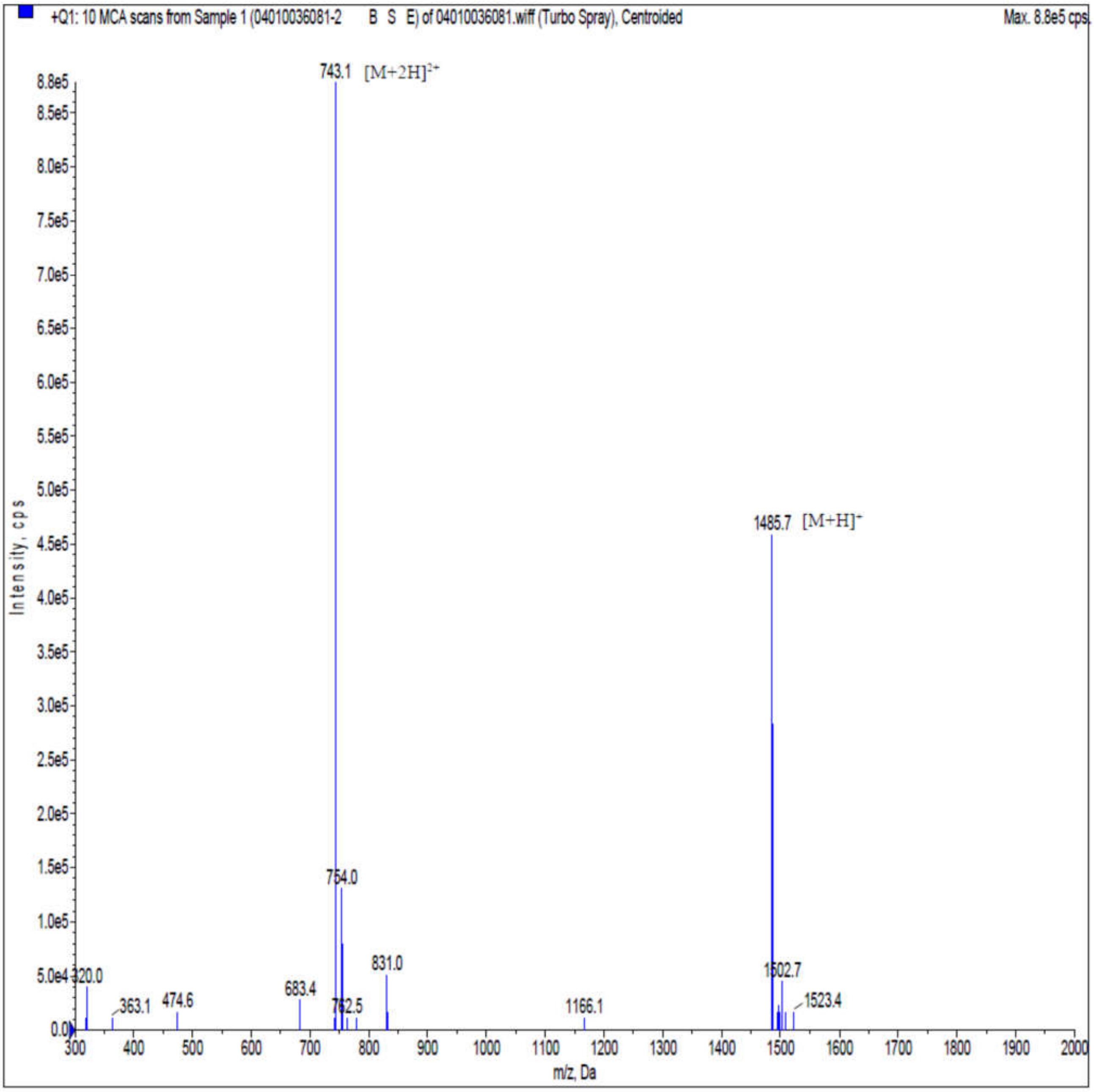

Figure S3. MS spectrum of NOTA-PSMA-CM
